# Determinants of polyunsaturated fatty acid concentrations in erythrocytes of pregnant Japanese women from a birth cohort study: study protocol and baseline findings of an adjunct study of the Japan environment & Children’s study

**DOI:** 10.1186/s12199-017-0636-5

**Published:** 2017-04-04

**Authors:** Shoji Saito, Terue Kawabata, Nozomi Tatsuta, Fumiko Kimura, Teruo Miyazawa, Satoshi Mizuno, Hidekazu Nishigori, Takahiro Arima, Yasuo Kagawa, Kouichi Yoshimasu, Kanami Tsuno, Yuki Ito, Michihiro Kamijima, Kunihiko Nakai, Nobuo Yaegashi, Miyagi Study Group of Japan Environment & Children’s Study

**Affiliations:** 1 Department of Gynecology and Obstetrics, Tohoku University Graduate School of Medicine, Sendai, Japan; 2 Department of Obstetrics and Gynecology, Yamagata Prefectural Central Hospital, Aoyagi 1800, Yamagata, 990-2292 Japan; 3 Faculty of Nutrition, Kagawa Nutrition University, Saitama, Japan; 4 Department of Development and Environmental Medicine, Tohoku University Graduate School of Medicine, Sendai, Japan; 5 Food and Biodynamic Chemistry Laboratory, Graduate School of Agricultural Sciences, Tohoku University, Sendai, Japan; 6 Department of Human Health and Nutrition, Shokei Gakuin University Graduate School, Natori, Japan; 7 New Industry Creation Hatchery Center, Tohoku University, Sendai, Japan; 8 Department of Informative Genetics, Tohoku University Graduate School of Medicine, Sendai, Japan; 9 Department of Hygiene, School of Medicine, Wakayama Medical University, Wakayama, Japan; 10 Department of Occupational and Environmental Health, Nagoya City University Graduate School of Medical Sciences, Nagoya, Japan

**Keywords:** Birth cohort study, Docosahexaenoic acid, Eicosapentaenoic acid, Japan environment and children’s study, N-3 polyunsaturated fatty acids

## Abstract

**Background:**

N-3 polyunsaturated fatty acids (n-3 PUFA) may have multiple beneficial effects on the outcome of pregnancy, maternal health and child development. The present study introduced the protocol of a birth cohort study to examine the beneficial effects of n-3 PUFA status in pregnant Japanese women as an adjunct study of the Japan Environment and Children’s Study (JECS).

**Methods:**

The JECS participants in the coastal areas of Miyagi Prefecture were further invited to participate in this adjunct study, and 1,878 pregnant women were enrolled prior to delivery. Their n-3 PUFA status was evaluated with fatty acid profiles in erythrocytes of maternal blood collected from 1,623 mothers at 24–30 weeks of gestation and cord blood from 1,505 deliveries.

**Results:**

The baseline results, including comprehensive data on the fatty acid status and determinants affecting the PUFA status, were analyzed. In stepwise multivariate analyses, the cord blood docosahexaenoic acid (DHA) level was found to be significantly influenced by the DHA level in maternal blood, the child’s sex, and the gestational period. The maternal DHA level was influenced by fish intake, maternal age, and the prepregnancy body mass index. While cord blood eicosapentaenoic acid (EPA) was influenced by maternal EPA, fish intake, and season at birth, additional factors such as maternal education, household income, and smoking habits affected the maternal EPA content.

**Conclusion:**

Further studies are warranted to clarify the nutritional impacts of n-3 PUFA in pregnant Japanese women of the cohort study.

## Background

N-3 polyunsaturated fatty acids (n-3 PUFA) are of critical importance during pregnancy and early life. Several observational studies and randomized trials showed the beneficial effects of n-3 PUFA during pregnancy and/or lactation for normal fetal and postnatal child growth and development such as cognitive performance, attention, information processing, and visual acuity [1–6]. Furthermore, PUFA during early life may modify fetal programming that influences the susceptibility and individual risk for developing metabolic diseases throughout life [7]. Supplementation of n-3 PUFA during pregnancy reduces the incidence of early preterm birth [6]. The maternal n-3 PUFA status has also been shown to be associated with maternal outcomes such as psychological distress [8] and postpartum depression [9]. Maternal obesity and excessive weight gain during pregnancy are reported to be associated with an adverse fatty acid profile such as higher concentrations of saturated fatty acids, monounsaturated fatty acids, and n-6 PUFA in plasma of pregnant women [10]. Although the causal relation with fatty acid intake remains undetermined, these findings suggest the importance of the quality of the fatty acid profile in pregnant women.

Since fish and seafood are rich in n-3 PUFA, fish consumption is the primary route for the intake. Therefore, the n-3 PUFA level in the blood of fish-eating populations is usually high. However, most of the above studies indicating beneficial effects have been conducted in the USA or other Western countries where most women have relatively low intakes of n-3 PUFA [11]. In this context, it should be examined whether n-3 PUFA is still beneficial in a population that consumes a large amount of fish and seafood.

Furthermore, fish are also contaminated with several toxic chemicals such as methylmercury and persistent organic pollutants (POPs) through bioaccumulation in the aquatic food chain. Prenatal exposure to these chemicals induces several adverse effects, including fetal growth impairment [12, 13] and delayed neurobehavioral development [14, 15]. These findings suggest the necessity of mutual adjustment of both factors, and the balance of the beneficial and adverse effects of fish consumption should be considered to explore the nutritional benefits of n-3 PUFA in pregnancy for fish-eating populations, including Japanese women.

The PUFA status of the fetus and infant depends primarily on the maternal PUFA status and has to be ensured by placental [4] or breast milk transfer [16]. The maternal PUFA status is primarily dependent on maternal fish consumption, and several other factors have been shown to be related to the fatty acid status in pregnant women. These include the birth order [17], prepregnancy body mass index (BMI) [10], period of gestation [18], maternal gestational weight gain [10], smoking habit [11, 18], and PUFA supplementation [18]. In healthy adults, additional factors, including age [19–21], BMI [19], educational level [22, 23], income [22], race [20], alcohol use [20], and hormonal status [24] were also shown to affect the PUFA profile, and physical activity has been identified to be inversely associated in Japanese [21]. While eicosapentaenoic acid (EPA, 20:5n-3), docosahexaenic acid (DHA, 22:6n-3) and arachidonic acid (ARA, 20:4n-6) can be formed from the precursors α-linolenic acid (ALA, 18:3n-3) and linoleic acid (LA, 18:2n-6), respectively [25], several single nucleotide polymorphisms (SNPs) in the fatty acid desaturase gene cluster affect the PUFA profile in Japanese women [26]. A better knowledge of these factors could help to improve the PUFA status in pregnant women.

To clarify the beneficial effects of PUFA on maternal outcomes and child development in pregnant Japanese women, we designed a birth cohort study as an adjunct study of The Japan Environment & Children’s Study (JECS). JECS is an ongoing national large prospective cohort study to evaluate the impacts of various environmental factors on child health and development. As the adjunct study, we added the determination of PUFA levels in maternal and cord blood erythrocytes and maternal breast milk to evaluate the nutritional advantages of maternal PUFA intake. In this interim report, we present the study protocol, participants’ characteristics, the baseline data and the determinants of PUFA status in maternal and cord blood erythrocytes.

## Methods

### Study population

**Fig. 1:**
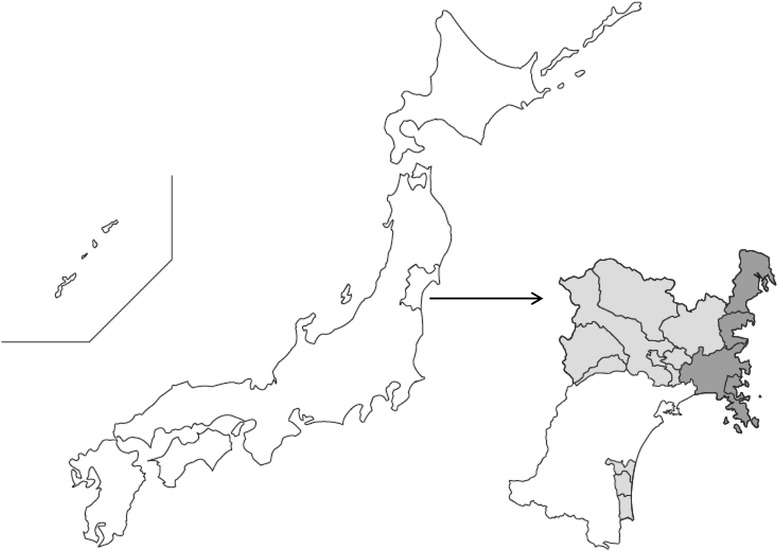
Study area of JECS (light gray) and of the adjunct study (dark gray) in Miyagi Prefecture

JECS, funded by the Ministry of the Environment (MOE), is a large, nationwide longitudinal birth cohort study with follow-up programs to examine health from the early stages of pregnancy until the participating child reaches 13 years of age. Details of the JECS recruitment and sampling strategy as well as data collection procedures have been previously described [27]. Of the 15 regional centers of JECS, the Miyagi Regional Center covers 14 local government areas for the main study in Miyagi Prefecture (Fig. 1). In this adjunct study, the participants in the resident registers of 4 local governments, Kesennuma, Minamisanriku, Onagawa, and Ishinomaki, were further recruited to participate in the adjunct study when they were in the first trimester. The participants were asked to provide written informed consent for the JECS main study, and next for the adjunct study. Although the JECS recruitment period was from January 2011 until March 2014 for the main study, it was from December 2011 until October 2013 for the adjunct study. The eligibility criteria of the adjunct study were the same as for JECS: (1) residence in the study areas, (2) expected delivery date between 1 August 2011 and mid-2014, and (3) capable of participating in the study without difficulty [27].

**Fig. 2:**
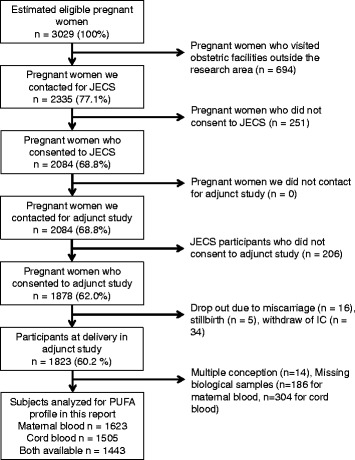
Enrollment in JECS and the adjunct study

Single mutation and/or genomewide analyses were planned in JECS, and the storage of biological samples for genetic analyses was included in the informed consent [27], though genetic analysis itself was not originally included in the informed consent. Therefore, the participants in this adjunct study were further asked after delivery to provide written informed content for the analysis of single nucleotide polymorphisms, including desaturation and elongation of PUFA, using stored maternal and cord blood. Both JECS and this adjunct study were approved by the Ethics Committee of the Tohoku University Graduate School of Medicine.

### Study protocol

During pregnancy, self-administrated questionnaires were distributed to the pregnant women in their first and second trimesters, and at delivery in JECS [27, 28]. Questionnaires were also distributed to each family periodically after delivery. The JECS questionnaires during pregnancy included all information on basic characteristics and others, including questions about food frequencies, dietary supplement intakes, and physical activities using the short version of the International Physical Activity Questionnaire. The details of the questionnaires were described elsewhere [28]. Medical information was obtained from maternal and infant medical records by JECS, and the collected data were provided to this adjunct study through the data management system of JECS.

Additional procedures for the adjunct study were added to JECS only when they did not disturb the main study. We added the Center for Epidemiological Studies Depression (CES-D) scale to the questionnaire of the second trimester for screening depression. Maternal IQ was evaluated using the Raven standard progressive matrices [15] when the children were aged 7 months. We also added several neurobehavioral assessments for measurement of child neurobehavioral development [29]. These included the neonatal behavioral assessment scale at birth, Kyoto Scale of Psychological Development 2001 at 7 and 24 months, and Kaufman Assessment Battery for Children (K-ABC) at 42 months. Furthermore, the behaviors related to symptoms of attention deficit/hyperactivity disorder (ADHD) were also measured using teacher-rated scales when the children were in kindergarten or nursery school.

Maternal blood samples were obtained from the mothers at 24–30 weeks of gestation without fasting. Umbilical cord blood samples were obtained from the umbilical cord vein at birth. A vacuum sampling tube with EDTA-2Na as an anticoagulant was used for the blood sampling. These blood samples were transferred to the laboratory within 3 days at 4 °C, and centrifuged at 1600 *g* for 10 min to separate plasma and erythrocytes. The cells were washed twice with physiological saline, and the buffy coat was removed. The plasma and washed erythrocytes were stored at −80 °C until fatty acid determination. These procedures did not affect the concentrations of fatty acids [30].

### Analysis of erythrocyte fatty acid composition

Total erythrocyte lipid was extracted using isopropanol/chloroform according to the method of Rose and Oklander [31]. Briefly, distilled water with 0.1 mM EDTA-2Na was added to the cells and mixed. After 15 min, isopropanol was added slowly with mixing and allowed to stand for 1 h. After chloroform was added and mixed for 1 h, the lipid was transferred into chloroform-methanol 2:1 (v/v) and washed with distilled water with 0.9% KCl. After centrifugation at 1,600 × g for 5 min, the lipid extract obtained in the lower phase was methylated using methanol hydrochloride, and then gas-liquid chromatographic analysis of the fatty acid methyl ester compositions was conducted, as described previously [32].

### Statistical analyses

We obtained information from medical records and/or self-administred questionnaires. The medical record data were used primarily, whereas the information collected through questionnaire was used only as a fallback measure. The information included maternal age at blood collection, marital status (married/not married), educational background (<13 years/longer), household income (5 ranks), living together with parents (Y/N), smoking habits (never smoker/ex-smokers who quit before pregnancy confirmation/ex-smokers who quit after pregnancy confirmation/smokers during pregnancy), passive smoking (Y/N), alcohol consumption (Y/N), history of obstetrical/gynecological diseases, height and weight before pregnancy, parity (primiparae/multiparae), infertility treatment (Y/N), gestational age at blood collection and at birth, the child’s sex, type of delivery (transvaginal/caesarean section), and birth weight and height. Regarding the smoking habit, the same questionnaire was administered in both the first and second trimesters. We identified the participants as smokers or ex-smokers when they responded to the smoking question in either of the two questionnaires. Alcohol consumption status was divided into two categories in the questionnaire for the second trimester: non-drinkers (i.e., non-drinkers and ex-drinkers who quit drinking before or after confirmation of pregnancy) and drinkers (i.e., participants who continued drinking after confirmation of pregnancy).

In the FFQ, the subjects gave the frequency and portions of consumption for each item over the previous one month during the second trimester. The amounts of consumption were calculated from the frequencies and the portions given by the subjects individually; fish and seafood consumption was from the sum of 21 items, and meat consumption from that of 19 items.

The normality of continuous data was examined using the Shapiro-Wilk test. Since large skewed distributions were observed in the EPA content for maternal blood (skewness = 1.81) and cord blood (skewness = 3.48), EPA was used for analysis after logarithmic transformation. Intakes of fish and meat were also used for analysis after logarithmic transformation. The differences of basic characteristics between the participants of this adjunct study and the non-participants were analyzed using analysis of variance, the chi-square test, and the Fisher exact probability test.

For analysis of the determinants of PUFA profiles, those who had diabetes mellitus and gestational diabetes mellitus were excluded from further analyses because these pathological conditions might affect the metabolism and transport of PUFA between the placenta and fetal blood [4, 18, 19]. Those who had pregnancy-induced hypertension, hyperthyroidism, intrauterine growth restriction, and placental abruption were also excluded because of possible associations with delayed fetal development. Univariate associations between PUFA and other variables were examined using Pearson product-moment correlation coefficients (for continuous data) and ANOVA (for categorical data) as appropriate. The variables that were statistically significant in the univariate analysis at the *p <* 0.05 level were entered into exploratory multiple stepwise regression analyses. Other factors with strong a priori assumptions (e.g., a maternal smoking habit [33] and physical activity [21]) based on the findings of previous reports were also considered in the analysis of maternal blood. The Akaike information criteria were used in stepwise forward selection for determination of important factors. Participants with missing data for exposure variables were excluded. Variables were tested for collinearity with the variance inflation factor (VIF). All analyses, with two-sided *p* values, were carried out using the JMP software package version 7 · 0 (SAS Institute Inc., Cary, NC, USA).

## Results

### Registration and basic characteristics

Of the 9,217 JECS participants, 1,878 pregnant women joined in the adjunct study (Fig. 2). Since the number of maternal and child health handbooks issued by the local governments was 3,029 during the period of enrollment in the adjunct study, the coverage rate was approximately 62%. The numbers of participants who were included in the determination of factors influencing the PUFA profiles were 1,623 for maternal blood, and 1,505 for cord blood.

Characteristics of the mothers and their babies are presented in Table 1. The participants in the adjunct study were slightly older, and the rate of housewifes was higher than for the non-participants. More mothers who participated smoked, skipped breakfast, used some kind of supplementation, and were exposed to environmental tobacco smoke. Prepregnancy BMI, maternal body weight gain at delivery, gestational age and the number of alcohol drinkers were smaller among the participants. The percentage of low-birth-weight newborns (<2,500 g) was 9.8% in the participants in comparison with 7.8% in non-participants.

**Table 1:** Basic characteristics of the participants and the non-participants having live birth in the adjunct study of JECS

Variables	All JECS Participants		Participants of adjunct study Non-participants				
	n		n		n		*p*
Mothers with live birth	8873		1823		7050		
Age at delivery (yrs)	8823	30.7 ± 5.1	1817	31.0 ± 5.3	7006	30.6 ± 5.0	0.0022
Marital status at registration (Married, %)	8798	94.7	1807	94.3	6991	94.8	0.39
Maternal education (<13 yrs, %)	8746	52.9	1790	54.1	6956	52.3	0.27
Paternal education (<13 yrs, %)	8695	59.3	1778	62.9	6917	59.6	0.013
Household income (million Japanese Yen) (%)							
< 2	575	7.4	121	7.4	454	7.4	0.15
2 to <4	3111	39.9	694	42.6	2417	39.2	
4 to <6	2290	29.4	454	27.8	1836	29.8	
6 to <8	972	12.5	205	12.6	767	12.5	
≥ 8	842	10.8	157	9.6	685	11.1	
Living together with parents (Yes, %)	8835	46.7	1818	43.3	7017	47.5	0.0012
Smoking habits (%)							
Never smoked	4324	49.0	842	46.4	3482	49.6	0.034
Ex-smokers who quit before pregnancy	2099	23.8	440	24.2	1659	23.6	
Ex-smokers who quit after pregnancy	1752	19.8	377	20.8	1375	19.6	
Smokers during early pregnancy	657	7.4	157	8.6	500	7.1	
Passive smoking (Yes, %)	8850	66.4	1819	69.2	7031	65.7	0.0047
Alcohol consumption (Drinkers, %)	8767	2.5	1791	1.7	6976	2.7	0.013
Prepregnancy BMI (kg/m2)	8872	21.8 ± 3.6	1823	21.9 ± 3.9	7049	21.7 ± 3.6	0.019
Body weight gain at delivery (kg)	8822	10.8 ± 4.2	1813	10.4 ± 4.2	7009	10.9 ± 4.1	<0.0001
Physical activity (mets · min/week)	8766	480 (0–18400)	1792	480 (0–18400)	6974	480 (0–16400)	0.81
Obstetrical/gynecological disease (%)							
Diabetes mellitus	8866	0.73	1822	0.49	7044	0.80	0.16
Gestational diabetes mellitus	8866	1.5	1822	1.1	7044	1.6	0.11
Pregnancy-induce hypertension	8866	1.1	1822	2.4	7044	0.82	<0.0001
Hyperthyroidism	8866	0.43	1822	0.27	7044	0.47	0.23
Intrauterine growth restriction	8866	1.2	1822	1.4	7044	1.2	0.37
Placenta abruption	8866	0.20	1822	0.0	7044	0.26	0.035^a^
Parity (Primipara, %)	8783	39.4	1789	37.8	6994	39.8	0.12
Infertility treatment (Yes, %)	8814	4.6	1812	5.9	7002	4.3	0.0059
Fish intake (g/day)	8757	29.1 (0.8–919)	1788	1788 28.3 (1.2–612)	6969	29.4 (0.8–919)	0.11
Meat intake (g/day)	8762	54.6 (0.4–1740)	1789	55.5 (0.4–1581)	6973	54.3 (1.5–1740)	0.21
Supplemental use (%)							
Any kind	8774	38.8	1793	34.2	6981	40.0	<0.0001
EPA and/or DHA	8773	2.2	1792	2.5	5981	2.1	0.30
Breakfast skipper (Skipper, %)	8756	13.8	1788	14.8	6968	13.5	0.14
Housewife (Yes, %)	8742	44.0	1787	50.6	6955	42.3	<0.0001
Number of live births							
Singleton births (%)	8873	99.2	1823	99.2	7050	99.1	0.69
Gestational age at birth (day)	8857	274.1 ± 11.8	1819	273.6 ± 13.5	7038	274.3 ± 11.3	0.025
Preterm births (<37) (%)	8857	5.5	1819	5.4	7038	5.5	0.93
Sex (Male, %)	8865	51.0	1822	51.6	7043	50.9	0.35
Type of delivery (Transvaginal, %)	8847	82.2	1819	81.9	7028	82.2	0.74
Birth weight (g, Singleton births)							
All	8850	3046 ± 441	1818	3031 ± 479	7032	3050 ± 431	0.12
Male	4481	3095 ± 433	939	3076 ± 485	3580	3089 ± 433	0.43
Female	4289	3010 ± 427	876	2984 ± 468	3450	3008 ± 424	0.15
Low birth weight (<2500 g) (%)	8850	8.2	1818	9.8	7032	7.8	0.005

Mean ± SD or median (min-max)

^a^Fisher exact test

### PUFA status

The percentages by weight of fatty acids in maternal and cord blood erythrocytes are shown in Table 2. The DHA level was 7.4% in maternal blood erythrocytes, which roughly corresponded to the DHA levels that were previously reported in the general Japanese population [21, 26], and was higher than reported for Western countries [4, 20].

**Table 2:** Percentages by weight of fatty acid in maternal and cord blood erythrocytes

Fatty acid		Maternal blood (*n =* 1623)	Cord blood (*n =* 1505)
14:0		0.4 (0.2–1.2)	0.4 (0.0–0.9)
15:0		0.1 (0.0–0.5)	0.1 (0.0–0.3)
16:0		24.7 (20.4–30.4)	27.0 (23.1–32.3)
16:1n-7		0.3 (0.0–1.0)	0.4 (0.0–1.9)
18:0		15.7 (12.5–25.2)	18.0 (14.7–34.6)
18:1n-7		1.1 (0.0–1.7)	1.6 (0.1–2.3)
18:1n-9		12.8 (9.0–18.3)	10.0 (6.4–17.6)
18:2n-6	LA	9.1 (4.2–14.0)	4.2 (2.6–9.6)
18:3n-3	ALA	0.2 (0.0–2.7)	0.0 (0.0–0.6)
20:3n-6		1.3 (0.7–2.5)	2.4 (1.2–4.2)
20:4n-6	ARA	11.6 (6.6–16.2)	15.5 (9.1–20.0)
20:5n-3	EPA	0.7 (0.1–4.1)	0.3 (0.0–2.5)
22:0		1.2 (0.0–9.8)	1.0 (0.0–3.3)
22:4n-6		2.0 (0.2–4.3)	2.9 (0.8–5.2)
22:5n-3		1.9 (0.9–3.5)	0.7 (0.3–1.9)
22:6n-3	DHA	7.4 (3.2–11.0)	6.8 (3.1–10.7)
24:0		3.3 (0.9–6.8)	3.8 (2.9–5.5)
24:1n-9		4.1 (0.9–6.2)	2.9 (0.7–7.0)

Median (min-max)

### Determinants of PUFA profiles

The univariate associations of DHA, EPA, and ARA in maternal and cord blood with basic characteristics are shown in Table 3 for continuous data and Table 4 for categorical data. Maternal erythrocyte DHA and EPA levels were weakly correlated with maternal age and fish intake, whereas maternal ARA was inversely correlated with these factors, and positively correlated with meat intake. Maternal DHA and EPA were also associated with maternal education, household income, smoking, and passive smoking. The fatty acids in cord blood showed a high correlation with the corresponding fatty acids in maternal blood. Furthermore, cord blood erythrocyte EPA showed a similar association to that of maternal EPA. Cord blood DHA had a positive correlation with body weight gain during pregnancy, gestational age, and fish intake, whereas cord blood erythrocyte ARA was inversely correlated with gestational age and fish intake.

**Table 3:** Univariate associations between basic characteristics and PUFA levels in maternal and cord blood erythrocyte (Continuous data)

Variables	Maternal blood				Cord blood			
	n	DHA	log-EPA	ARA	n	DHA	log-EPA	ARA
Age at blood collection	1524	0.157 (<.0001)	0.151 (<.0001)	−0.113 (<.0001)	-	-	-	-
Age at delivery	-	-	-	-	1439	0.048 (0.068)	0.083 (0.0016)	−0.068 (0.010)
Prepregnancy BMI	1547	−0.033 (0.19)	−0.014 (0.59)	−0.019 (0.46)	1442	0.002 (0.93)	0.009 (0.72)	0.003 (0.92)
Body weight gain at blood collection	1544	0.022 (0.39)	0.068 (0.0076)	−0.058 (0.023)	-	-	-	-
Body weight gain at delivery	-	-	-	-	1439	0.097 (0.0002)	0.033 (0.21)	−0.028 (0.29)
Gestational age at blood collection	1544	0.005 (0.85)	0.0043 (0.87)	−0.013 (0.61)				
Gestational age at birth	-	-	-	-	1442	0.183 (<.0001)	0.005 (0.86)	−0.14 (<.0001)
Physical activity	1528	−0.021 (0.42)	−0.049 (0.056)	0.037 (0.16)	1432	−0.017 (0.53)	−0.025 (0.34)	0.034 (0.20)
Fish intake	1524	0.186 (<.0001)	0.233 (<.0001)	−0.149 (<.0001)	1428	0.109 (<.0001)	0.171 (<.0001)	−0.135 (<.0001)
Meat intake	1525	−0.040 (0.12)	−0.031 (0.23)	0.072 (0.0052)	1429	−0.043 (0.10)	−0.048 (0.070)	0.044 (0.87)
Each PUFA in maternal blood	-	-	-	-	1442	0.349 (<.0001)	0.625 (<.0001)	0.334 (<.0001)

Examined with Pearson correlation, r (p)

**Table 4:** Univariate associations between basic characteristics and PUFA levels in maternal and cord blood erythrocyte (Categorical data)

Variables	Maternal blood					Cord blood			
	n		DHA	EPA	ARA	n	DHA	EPA	ARA
Maternal education									
< 13		819	7.3 (3.2–11.0)	0.67 (0.11–2.96)	11.7 (7.3–14.5)	770	6.8 (3.3–10.5)	0.28 (0.02–2.54)	15.5 (9.0–19.9)
≥ 13		707	7.6 (4.1–10.7)	0.80 (0.16–4.10)	11.5 (6.6–16.2)	660	6.9 (3.1–10.7)	0.31 (0.03–2.30)	15.4 (9.7–20.0)
p			<.0001	<.0001	0.060		0.13	<.0001	0.11
Household income (million Japanese Yen)									
< 2		105	7.3 (4.3–10.3)	0.68 (0.25–2.05)	11.6 (8.1–14.0)	97	6.8 (3.9–9.5)	0.29 (0.08–2.30)	15.5 (9.0–19.9)
2 to <4		592	7.3 (3.2–11.0)	0.68 (0.14–3.00)	11.7 (7.3–14.6)	556	6.8 (3.1–10.5)	0.27 (0.03–2.54)	15.5 (10.9–20.0)
4 to <6		395	7.4 (3.9–10.5)	0.74 (0.11–2.40)	11.6 (6.9–14.1)	364	6.8 (3.4–10.7)	0.30 (0.02–2.04)	15.5 (9.7–18.4)
6 to <8		171	7.7 (4.4–10.7)	0.80 (0.29–4.10)	11.5 (8.3–16.2)	160	7.0 (3.9–9.8)	0.33 (0.07–1.71)	15.4 (10.9–18.7)
≥ 8		133	7.7 (4.8–10.1)	0.87 (0.31–3.44)	11.4 (7.1–14.5)	126	6.9 (3.3–9.1)	0.31 (0.08–1.38)	15.3 (10.7–19.6)
p			0.0005	<.0001	0.14		0.67	0.0004	0.33
Living together with parents									
Yes		661	7.5 (3.2–11.0)	0.75 (0.16–3.44)	11.6 (7.1–14.3)	629	6.8 (3.3–10.7)	0.29 (0.03–2.54)	15.4 (9.7–20.0)
No		883	7.4 (3.9–10.9)	0.70 (0.11–4.10)	11.6 (6.6–16.2)	811	6.8 (3.1–10.5)	0.29 (0.02–2.30)	15.5 (9.0–19.9)
p			0.11	0.081	0.28		0.81	0.082	0.074
Smoking habits									
Never smoked		698	7.5 (3.9–11.0)	0.75 (0.24–4.10)	11.6 (6.6–16.2)	654	6.8 (3.1–10.5)	0.29 (0.05–2.54)	15.4 (9.0–19.6)
Ex-smokers who quit before pregnancy		378	7.5 (4.3–10.3)	0.75 (0.11–2.88)	11.5 (7.2–14.3)	355	6.9 (3.3–9.4)	0.30 (0.02–1.60)	15.4 (9.7–18.6)
Ex-smokers who quit after pregnancy		330	7.2 (3.2–10.8)	0.67 (0.14–2.16)	11.6 (7.3–14.5)	303	6.8 (3.4–10.7)	0.26 (0.03–1.69)	15.5 (9.7–20.0)
Smokers during early pregnancy		137	7.1 (4.4–10.5)	0.68 (0.21–3.00)	11.7 (6.9–14.0)	126	6.8 (4.4–10.5)	0.27 (0.07–0.87)	15.5 (11.6–19.9)
p			<.0001	0.0010	0.16		0.75	0.0045	0.38
Passive smoking									
Yes		1075	7.3 (3.2–10.9)	0.70 (0.11–4.10)	11.6 (6.6–16.2)	1008	6.8 (3.1–10.5)	0.28 (0.02–2.54)	15.5 (9.0–20.0)
No		470	7.6 (3.9–11.0)	0.79 (0.25–2.68)	11.6 (7.2–14.6)	432	6.8 (3.3–10.7)	0.31 (0.05–1.38)	15.4 (10.2–18.6)
p			<.0001	<.0001	0.19		0.45	0.0014	0.11
Alcohol consumption									
Yes		29	7.6 (4.3–9.3)	0.75 (0.42–1.69)	11.2 (9.6–13.1)	26	7.0 (4.0–8.6)	0.27 (0.13–0.64)	15.3 (10.2–18.1)
No		1498	7.4 (3.2–11.0)	0.73 (0.11–4.10)	11.6 (6.6–16.2)	1405	6.8 (3.1–10.7)	0.29 (0.02–2.54)	15.5 (9.0–20.0)
p			0.78	0.50	0.12		0.91	0.87	0.72
Parity									
Primipara		574	7.4 (3.2–11.0)	0.72 (0.16–4.10)	11.6 (6.6–14.1)	525	6.8 (3.1–10.7)	0.30 (0.03–2.30)	15.4 (9.7–20.0)
Multipara		947	8.2 (3.9–10.7)	0.73 (0.11–3.44)	11.6 (6.9–16.2)	893	6.8 (3.3–10.5)	0.29 (0.02–2.54)	15.5 (9.0–19.9)
p			0.62	0.88	0.87		0.13	0.23	0.037
Supplemental use									
Any kind	Yes	523	7.5 (4.1–10.9)	0.75 (0.14–4.10)	11.6 (6.6–14.4)	474	6.8 (3.3–10.1)	0.30 (0.02–1.69)	15.5 (10.7–18.6)
	No	1006	7.4 (3.2–11.0)	0.72 (0.11–2.96)	11.6 (6.9–16.2)	959	6.8 (3.1–10.7)	0.29 (0.03–2.54)	15.5 (9.0–20.0)
p			0.012	0.042	0.27		0.44	0.60	0.37
EPA and/or DHA	Yes	43	7.3 (4.9–10.1)	0.74 (0.25–1.95)	11.6 (9.4–14.4)	37	6.9 (5.4–8.7)	0.30 (0.13–0.55)	15.5 (12.6–18.3)
	No	1485	7.4 (3.2–11.0)	0.73 (0.11–4.10)	11.6 (6.6–16.2)	1395	6.8 (3.1–10.7)	0.29 (0.02–2.54)	15.4 (9.0–20.0)
p			0.87		0.72	0.91	0.95	0.93	0.38
Breakfast skipper									
Skipper		235	7.3 (4.3–10.5)	0.70 (0.25–2.96)	11.7 (8.3–14.0)	222	6.8 (3.4–9.2)	0.29 (0.08–1.69)	15.7 (9.7–20.0)
Eater		1289	7.4 (3.2–11.0)	0.73 (0.11–4.10)	11.6 (6.6–16.2)	1206	6.8 (3.1–10.7)	0.29 (0.02–2.54)	15.4 (9.0–19.6)
p			0.028	0.18	0.40		0.97	0.65	0.010
Housewife									
Yes		771	7.4 (3.2–11.0)	0.70 (0.11–4.10)	11.7 (7.2–14.6)	717	6.8 (3.3–10.7)	0.28 (0.03–2.54)	15.5 (9.0–20.0)
Yes		752	7.4 (4.1–10.9)	0.76 (0.16–3.44)	11.6 (6.6–16.2)	710	6.8 (3.1–10.5)	0.30 (0.02–2.04)	15.4 (9.7–19.6)
p			0.17	0.0001	0.41		0.53	0.10	0.15
Sex of child									
Male		794	7.4 (3.2–10.8)	0.74 (0.14–3.44)	11.6 (6.6–14.6)	735	6.9 (3.1–10.5)	0.29 (0.05–2.54)	15.2 (9.7–20.0)
Female		749	7.4 (3.6–11.0)	0.70 (0.11–4.10)	11.6 (6.9–16.2)	707	6.7 (3.3–10.7)	0.29 (0.02–1.71)	15.8 (9.0–19.9)
p			0.045	0.0047	0.24		0.0002	0.49	<.0001
Type of delivery									
Transvaginal		1318	7.4 (3.2–11.0)	0.72 (0.11–3.44)	11.6 (6.6–14.5)	1243	6.8 (3.1–10.7)	0.29 (0.02–2.54)	15.4 (9.0–20.0)
Caesarean		226	7.5 (4.6–10.7	0.76 (0.14–4.10)	11.6 (7.9–16.2)	199	6.6 (3.9–9.8)	0.31 (0.07–1.36)	15.7 (10.9–19.9)
p			0.36	0.16	0.81		0.012	0.09	0.016
Season at blood collection									
Spring		357	7.3 (3.6–10.5)	0.67 (0.16–2.16)	11.6 (8.8–14.1)	-	-	-	-
Summer		442	7.4 (4.4–10.9)	0.69 (0.14–2.70)	11.6 (7.1–14.2)	-	-	-	-
Autumn		373	7.4 (3.2–10.8)	0.77 (0.11–4.10)	11.7 (6.6–14.3)	-	-	-	-
Winter		355	7.5 (3.9–11.0)	0.76 (0.23–2.68)	11.5 (6.9–16.2)	-	-	-	-
p			0.046	0.046	0.046	-	-	-	-
Season at delivery									
Spring		-	-	-	-	355	6.7 (4.0–9.8)	0.28 (0.08–1.13)	15.5 (10.5–20.0)
Summer		-	-	-	-	328	6.8 (4.0–9.2)	0.26 (0.02–2.04)	15.4 (11.9–19.9)
Autumn		-	-	-	-	404	7.0 (3.1–10.7)	0.33 (0.04–2.54)	15.6 (9.0–19.6)
Winter		-	-	-	-	355	6.9 (3.3–10.5)	0.30 (0.05–1.15)	15.4 (10.7–18.6)
p							0.10	<.0001	0.98

Median (min-max)

Compared with ANOVA

EPA was analyzed after log-transformation

Variables having significant associations in these univariate analyses and some other factors such as a maternal smoking habit and physical activity were included in multiple stepwise regression analyses. The best regression models are shown in Table 5 for maternal blood and Table 6 for cord blood. Maternal erythrocyte DHA was associated positively with fish intake and maternal age, and this fatty acid was decreased in smokers. Maternal EPA was associated positively with fish intake, maternal body weight gain, and age. This fatty acid increased in the autumn and in the participants having the highest income and higher educational level. In contrast, maternal ARA was associated inversely with fish intake, body weight gain, and maternal age. Meat intake was also associated positively with maternal ARA. In cord blood analyses, all fatty acids were potently associated with corresponding fatty acids in maternal blood. Cord blood DHA was associated with maternal body weight gain and gestational age, and the level of this fatty acid was higher in male newborns. However, there was no association between cord blood DHA content and maternal fish intake. In contrast, cord blood EPA was positively associated with fish intake, and a higher concentration was observed in the autumn. The cord blood ARA concentration was higher in female newborns and in mothers who skipped breakfast. This fatty acid was negatively associated with gestational age and fish intake.

**Table 5:** Multiple stepwise regression analyses for determinants of PUFA levels in maternal blood erythrocytes

	DHA (*n =* 1376)			log-EPA (*n =* 1374)			ARA (*n =* 1504)		
Adjusted model R^2^	0.072			0.122			0.056		
Variables	B	Standardized β	p	B	Standardized β	p	B	Standardized β	p
Maternal age at blood collection	0.025	0.12	<.0001	0.0034	0.090	0.0007	−0.020	−0.089	0.0005
Maternal education (<13 yrs)	0.056	0.049	0.078	0.019	0.10	0.0005	-		
Household income									
< 2 (million Japanese Yen)	reference			reference			-		
2 to <4	−0.084	−0.046	0.098	−0.021	−0.066	0.014	-		
4 to <6	−0.092	−0.046	0.11	−0.013	−0.034	0.19	-		
6 to <8	0.11	0.042	0.16	0.0079	0.018	0.54	-		
≥ 8	0.073	0.027	0.38	0.041	0.086	0.0034	-		
Smoking habits									
Never smoked	reference			reference			-		
Ex-smokers before pregnancy	−0.084	0.088	0.10	0.014	0.059	0.13	-		
Ex-smokers after pregnancy	−0.070	−0.048	0.23	−0.010	−0.038	0.34	-		
Smokers	−0.17	−0.10	0.032	−0.015	−0.049	0.27	-		
Body weight gain	-			0.0057	0.091	0.0004	−0.027	−0.072	0.0044
Fish intake	0.43	0.16	<.0001	0.094	0.20	<.0001	−0.57	−0.21	<.0001
Meat intake	-			-			0.58	0.17	<.0001
Sex (male)	0.055	0.049	0.061	0.0091	0.046	0.068	-		
Season at blood collection									
Spring	reference			reference			-		
Summer	−0.067	−0.043	0.17	−0.021	−0.076	0.012	-		
Autumn	0.051	0.031	0.32	0.032	0.11	0.0003	-		
Winter	0.087	0.052	0.097	0.028	0.097	0.0017	-		

Variables included in the model are indicated

**Table 6:** Multiple stepwise regression analyses for determinants of PUFA levels in cord blood erythrocytes

	DHA (*n =* 1379)			log-EPA (*n =* 1247)			ARA (*n =* 1365)		
Adjusted model R^2^	0.165			0.348			0.171		
Variables	B	Standardized β	p	B	Standardized β	p	B	Standardized β	p
PUFA level in maternal blood	0.31	0.34	<.0001	0.32	0.54	<.0001	0.39	0.31	<.0001
Household income									
< 2 (million Japanese Yen)	-			reference			-		
2 to <4	-			−0.022	−0.058	0.017	-		
4 to <6	-			0.008	0.019	0.45	-		
6 to <8	-			0.016	0.031	0.23	-		
≥ 8	-			−0.013	−0.022	0.40	-		
Smoking habits									
Never smoked	-			reference			-		
Ex-smokers before pregnancy	-			0.006	0.020	0.56	-		
Ex-smokers after pregnancy	-			−0.014	−0.046	0.19	-		
Smokers	-			−0.003	−0.007	0.86	-		
Body weight gain	0.016	0.064	0.011	-			-		
Fish intake	-			0.036	0.065	0.0061	−0.26	−0.079	0.0016
Breakfast skipper (Yes)	-			-			0.13	0.065	0.0088
Gestational age	0.020	0.18	<.0001	-			−0.024	−0.15	<.0001
Sex (male)	0.098	0.096	0.0001	-			−0.26	−0.18	<.0001
Season at birth									
Spring	-			reference			-		
Summer	-			−0.032	−0.095	0.0008	-		
Autumn	-			0.073	0.22	<.0001	-		
Winter	-			−0.023	−0.068	0.017	-		

Variables included in the model are indicated

## Discussion

In the present report, we described the protocol and the characteristics of the participants in a birth cohort study to examine the beneficial effects of n-3 PUFA on maternal health and child development. The number of participants in the adjunct study was sufficient for the above purposes. By adding several measurements to JECS, maternal health and child development could be observed in this adjunct study. In particular, the KSPD and K-ABC were expected to be useful to examine child development. The KSPD seems to be equivalent to the Bayley Scale of Child Development, which has been utilized in many epidemiological studies. The K-ABC has been used to demonstrate the beneficial effects of fish oil supplementation during pregnancy and lactation on child intelligence at the age of 4 years [1] and the adverse effects of prenatal PCB exposure in Japan [15]. Furthermore, this is the first study that connects an environmental measurement system including methylmercury and POPs that may be intaken through fish consumption. These unique features thereby can help us to examine the risks and benefits of fish consumption by comparing the effects of PUFA status and toxicant exposure on child development.

In the baseline data of this birth cohort study, we searched for dietary, anthropometric, and lifestyle determinants of DHA, EPA and ARA content in erythrocytes of maternal blood and cord blood. As expected, fish consumption was the primary determinant to increase the contents of DHA and EPA, and to decrease that of ARA in maternal blood. Similarly, fish consumption also increased EPA content, and decreased ARA content in cord blood, however, it did not affect the DHA content in cord blood. Although the explanation of this difference is not known, DHA incorporation into erythrocyte membrane is slow in individuals with higher DHA content, and it is supposed that erythrocyte membrane DHA content might be regulated [34]. A similar regulation may affect the transfer of DHA across the placenta. While it was reported that fish consumption increased in the elder population in Japan [21], age was still an independent determinant for all fatty acids even after the adjustment of covariates as reported previously [19, 35]. The effect of maternal smoking on the level of DHA in maternal red blood cells is controversial. Studies of pregnant German women [18] and schizophrenic patients [2] showed that smoking status predicted lower levels of DHA, but other studies did not confirm the effect of smoking [19, 21]. In the present study, maternal smoking was found to decrease the content of DHA and EPA in maternal blood. One possible explanation is that non-smokers ate more oily fishes than smokers [36]. Other possibilities included alteration in the metabolism and synthesis of fatty acids and oxidative destruction of highly unsaturated fatty acids after exposure to cigarette smoke. Interestingly, though, maternal smoking had no influence on PUFA profiles in cord blood in the present study. Seasonal changes in the content of EPA were observed both in maternal blood and cord blood. Increased consumption of oily fishes during autumn was probably related to the increased level of EPA. Similarly, the participants with higher household incomes had higher EPA content in maternal blood, suggesting a difference in the kind of consumed fishes dependent on the economic situation. Supplemental use of PUFA had no effect on the contents in maternal blood. Although the exact dose of PUFA supplementation was not inquired about in the questionnaire, a possible explanation is that the dose and frequency of PUFA supplementation might be too small. These above inferences must be evaluated through future studies including a quantitative FFQ. While a Japanese study indicated that physical activity was inversely related to the concentrations of DHA and EPA in the general population [21], physical activity was not identified as a determinant in pregnant women. Although the maternal ARA level was associated with skipping breakfast, the mechanism of this is currently unknown.

Basically, ARA and DHA are sourced both from the diet and endogenously from desaturation and elongation of their dietary precursors. Furthermore, several SNPs in the desaturase gene cluster affect the proportions of PUFA and long chain PUFA in human blood [26]. Since sex hormones also influence the activity of desaturase and elongase enzymes in women [24], the sex differences observed in the contents of cord blood DHA and ARA in the present study were possibly due in part to hormonal differences between female and male newborns. Genomic and hormonal examinations are currently under investigation in this adjunct study.

The present study has several limitations. First, it was a single-center study that was performed in coastal areas of the Tohoku region, where the fish consumption seems to be high. Second, soon after JECS started in January 2011, the Great East Japan earthquake and subsequent Tohoku tsunami occurred in the research areas on March 11, 2011. The registration of this adjunct study was started in October 2011; however, the life of most participants might be still influenced by the disaster to a greater or lesser extent. In particular, seafood consumption in these coastal areas might have been temporarily decreased due to harmful rumors about food safety and radionuclide contamination and damage to many harbors and fish markets [37]. In contrast, the strengths of the study include the relatively large number of participants and measurements of fatty acids in biological materials. Furthermore, this study was designed to cooperate with the national JECS study, in which risk analyses for several toxic substances such as POPs, heavy metals, and various other chemical exposures were performed simultaneously [27]. Since some toxicants are ingested through the consumption of seafood [38], this cooperation allowed us to examine the nutritional effects of n-3 PUFA while considering exposures to toxic contaminants derived from marine products as mentioned above. To our knowledge, this is the first birth cohort study to examine both the nutritional and toxic aspects of fish consumption in Japan.

## Conclusion

A birth cohort was established to examine the beneficial effects of PUFAs on maternal health and child development as an adjunct study of JECS. Currently child development is being monitored using several psychological and behavioral approaches. Since this adjunct study connects environmental measurement systems through JECS, this unique feature can thereby be used to examine both the beneficial and risk aspects of fish consumption simultaneously. Based on the baseline data, the determinants of PUFA status in maternal and cord blood were examined and, as expected, maternal fish consumption was a potent factor. Considering that the fish consumption of Japanese has been decreasing over the past decade according to National Health and Nutrition Surveys, understanding of non-dietary determinants will be of importance to improve the maternal status of PUFA. If the optimum PUFA level during pregnancy can be defined via this birth cohort study, better knowledge of the factors determining the levels of PUFAs in maternal and cord blood will help to improve the PUFA status in mothers and newborns.

## Abbreviations

ADHD: Attention deficit/hyperactivity disorder
ALA: α-linolenic acid
ARA: Arachidonic acid
BMI: Body mass index
CES-D: The center for epidemiological studies depression
DHA: Docosahexaenoic acid
EPA: Eicosapentaenoic acid
JECS: Japan Environment and children’s study
K-ABC: Kaufman assessment battery for children
LA: Linoleic acid
MOE: Ministry of the environment
n-3 PUFA: N-3 polyunsaturated fatty acids
n-3 PUFA: N-3 polyunsaturated fatty acids
POPs: Persistent organic pollutants
SNPs: Single nucleotide polymorphisms
